# Castable Bulk Metallic Glass Strain Wave Gears: Towards Decreasing the Cost of High-Performance Robotics

**DOI:** 10.1038/srep37773

**Published:** 2016-11-24

**Authors:** Douglas C. Hofmann, Raul Polit-Casillas, Scott N. Roberts, John-Paul Borgonia, Robert P. Dillon, Evan Hilgemann, Joanna Kolodziejska, Lauren Montemayor, Jong-ook Suh, Andrew Hoff, Kalind Carpenter, Aaron Parness, William L. Johnson, Andrew Kennett, Brian Wilcox

**Affiliations:** 1 Engineering and Science Directorate, Jet Propulsion Laboratory, California Institute of Technology, 4800 Oak Grove Dr. Pasadena CA 91109, USA; 2 Keck Laboratory of Engineering Sciences, California Institute of Technology, 1200 E. California Blvd., Pasadena CA 91125, USA

## Abstract

The use of bulk metallic glasses (BMGs) as the flexspline in strain wave gears (SWGs), also known as harmonic drives, is presented. SWGs are unique, ultra-precision gearboxes that function through the elastic flexing of a thin-walled cup, called a flexspline. The current research demonstrates that BMGs can be cast at extremely low cost relative to machining and can be implemented into SWGs as an alternative to steel. This approach may significantly reduce the cost of SWGs, enabling lower-cost robotics. The attractive properties of BMGs, such as hardness, elastic limit and yield strength, may also be suitable for extreme environment applications in spacecraft.

Precision gear systems, like those required in space applications and in advanced robotics, often require specially manufactured components with specialized performance characteristics. Among the desirable properties for space are high torque capacity, precision positioning, low mass, compact designs and extreme-environment operations. These requirements have resulted in the development of strain wave gears (SWG), also known as harmonic drive (HD) gears, which have made a significant impact in robots, machine tools and in spacecraft^1^. Invented in 1959, SWGs were first used in aerospace and defense applications and eventually accepted into spaceflight in 1971 as a component in the Apollo 15 lunar roving vehicle for NASA^1^,^2^. Since then, SWGs have been used widely by NASA for space telescopes (including Hubble) and rovers (including both Spirit and Opportunity from the Mars Exploration Rover mission). Currently proposed NASA missions to so-called “icy bodies,” consisting of moons such as Europa and Titan as well as comets and asteroids, will require advanced gear systems that can operate at extremely low temperatures. SWGs offer significant advantages over conventional gear systems, including high positioning accuracy with zero backlash, reduced size, reduced weight and increased reduction ratios^3^. These properties make them an attractive gear choice for high-performance applications, such as space and defense, but high cost due to ultra-precise manufacturing limits widespread use in lower cost, consumer-grade robotics. Due to the ultra-precise machining required of the components of SWGs, reducing their cost is challenging and material choices are limited, which has resulted in the vast majority of SWGs being made from steel. Although some attempts have been made to integrate low-cost designs and low-density materials into SWGs, the vast majority are still made from steel. For example, the outer spline of the SWG has been replaced with low density materials, such as aluminum, that have been coated with hard materials to improve the wear performance of aluminum^1^. Some research has focused on changing the flexspline material by using carbon-fiber/steel composites, high-strength polymers or a variety of metal alloys^4^,^5^. As such, research in SWGs primarily focuses on how to (1) lower cost, (2) achieve better performance in the intended environment, (3) lower mass, (4) integrate new materials and (5) make smaller drives.

The operation of a steel SWG is shown in Fig. 1. Although SWGs can be constructed using a variety of geometries, the three components of a standard *cup-type* SWG are shown disassembled in Fig. 1a, for a CSF-8 purchased from Harmonic Drive Systems, Inc., Tokyo, Japan. They are (1) a stiff outer spline, also called a circular spline, with internal gear teeth, (2) a thin-walled flexspline with external teeth numbering two less than the outer spline, and (3) an elliptical wave generator with steel ball bearings confined in an elliptical race by a steel band. When assembled, the wave generator forces the wall of the flexspline to expand and engage the teeth of the outer spline. The output torque is generally provided by the base of the flexspline while the outer spline stays fixed. The typical operation of a SWG is shown schematically in Fig. 1c. The wave generator forces the teeth on the flexspline to engage the outer spline and when the wave generator is rotated, the flexspline elastically deforms to maintain contact. After a 180 degree rotation, the flexpline has moved by one tooth relative to the outer spline. After a full rotation, the flexspline and the outer spline have been offset by two teeth. Unlike spur and planetary gears, the reduction ratio of a SWG is not a function of the size of the gears, but rather by the number of teeth. The reduction ratio, *i*, which is defined as the ratio of the input speed to the output speed is:


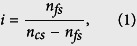


where *n*_*cs*_ is the number of teeth in the outer spline (or circular spline) and *n*_*fs*_ is the number of teeth in the flexspline. Using this equation a SWG can have reduction ratios that range from 30:1 to 320:1. Steel SWGs can be purchased in dimensions ranging from 20–300 mm in outer diameter^1^. SWGs can be run with extremely high efficiencies compared to other gear systems and are also designed to have low friction in the gear teeth. Due to the design of the teeth, the contact is almost purely radial, preventing much of the frictional wear that occurs in other gear systems, even though significant wear is often seen in failed SWGs^6^. As such, much effort has been spent designing the tooth profile for SWGs, which has been optimized into an S-shaped profile^3^.

Figure 1d shows a schematic of the load-life curve for a SWG. Although SWGs can produce a large torque ratio, they do this at the expense of the flexspline, the thin machined steel cup that transmits torque between the input and the outer spline. Figure 1d is a load torque versus number of cycles plot (which can be thought of as a stress-life curve) that is used to predict the operational conditions of the SWG. In general, the failure of the SWG is bounded at high load torques by ratcheting, which is described by the teeth of the flexspline slipping across the teeth of the wave generator, producing a condition known as “dedoidal.” A ratcheting event typically deforms the gear teeth on either the flexspline or the outer spline and renders the SWG inoperable. At high operational loads but below the ratcheting torque, the SWG will eventually fail due to fatigue of the flexspline. At loads below the fatigue strength of the flexspline, failure will normally occur due to the failure of the bearings in the wave generator. Based on these stress-life curves for SWGS, it is apparent that the flexspline must be designed from a high-performance metal alloy that has a combination of properties that are specifically suited to this unique application. For spacecraft uses, the alloy must also survive extreme cold and, in some cases, operation without lubricant. Some effort has been made designing new SWGs for space applications, including the use of coated aluminum in the outer spline and wave generator, and new compact designs to reduce mass^1^,^6^,^7^. However, nearly all of the SWGs used by NASA in spacecraft are lubricated steel.

Due to the loads on the flexspline, very few materials are suitable for SWGs. The flexpline must be able to be machined into a very thin wall to allow for flexing at reasonable stresses, it must be hard enough to avoid wear degradation in the teeth, it must be tough enough to avoid ratcheting and it must be exceptionally fatigue resistant. Due to these constraints, the vast majority of SWGs are manufactured from steel (typically from alloys such as 439 for conventional use and 304 L or 15–5 precipitation hardened stainless steels for space use). High cost is driven by the complex machining required of the thin flexspline, which is estimated to be half of the total cost of manufacturing the SWG. To illustrate this, consider a CSG-20 and CSF-8 commercial SWGs from Harmonic Drive Systems, Inc., with flexspline outer diameters of approximately 50 mm and 20 mm, respectively. For the larger flexspline, the minimum mass of the steel billet used to machine the flexspline is 463 g whereas the final machined mass is 28 g, resulting in a 94% scrap rate during the machining process. For the smaller flexspline, the minimum mass of the steel billet is 45 g and the final flexspline is 3 g, resulting in a 93% scrap rate during the machining. In addition, the wall thickness in the 20 mm diameter flexspline is only 125 μm at the root of the teeth, which demonstrates both the difficulty in machining this part as well as the limitations in minimum size that can be manufactured using conventional machining. This is the primary reason why smaller, compact SWGs don’t utilize the cup-flexspline geometry.

SWGs are a critical component of many robotic systems but widespread use is stifled by their cost. It is not uncommon that a substantial fraction of the cost of a robotic system is in the SWGs. Developing a technology to decrease the cost of manufacturing a SWG not only decreases the cost of existing systems that use these gears but also enables a whole new class of lower-cost robots that could utilize them. This would have a profound impact particularly on humanoid robotics, which typically require SWGs in the limbed joints to enable precise motion. To decrease the cost of SWGs, the complex metal components could be cast into a near-net or net-shape, however, the material must not significantly compromise the performance. Many attempts have been made to cast or rapidly fabricate SWG flexsplines, including casting against a die tooling, injection molding and additive manufacturing. Most of these efforts have been done with plastics, however, due to their lower viscosity and melting temperatures^8^,^9^. Bulk metallic glasses (BMGs), also known as amorphous metals, are a distinctive class of engineering materials that have many properties attractive for implementation into SWGs. BMGs are multi-component alloys (using four or more elements) that are designed around deep eutectics such that when they are cooled rapidly from the melt they form a non-crystalline microstructure. This imparts unique properties compared with crystalline metals, including high strength, high wear resistance, high hardness, and large elastic strain limit of^10^,^11^,^12^,^13^. Importantly, the low melting temperature of BMGs also allows them to be processed using injection-molding technology similar to plastics^14^. Moreover, BMGs have been developed with the density of titanium alloys (4.5–5.0 g/cm^3^) but with much higher hardness (500 vs. 350 Vickers) and elastic limit (2% vs. 1%)^15^. Recently, it has been demonstrated that BMGs can be cast into extremely complex compliant mechanisms, with feature sizes less than 1 mm^14^. The combination of ultra-high strength and large elastic strain limit resulted in performance that was typically twice as good as titanium and four times better than steel^14^. BMGs based in systems like Ti, Zr, Cu and Ni can have yield strengths up to 2 GPa with a 2% elastic limit caused by their low Young’s modulus (<100 GPa in most alloys). Compared to the steel currently used in SWGs, most BMGs are corrosion resistant (eg. they do not rust) and are non-ferromagnetic.

BMGs may seem like a natural choice for use in a SWG flexspline, but there are several reasons identified in the literature that may preclude such a use. One issue is the net-shaped casting of the flexspline, which contains both a thin wall and external gear teeth. Although complex parts made from BMGs have been demonstrated in the literature, a robust commercial process for casting a part with the combined features of the flexspline has not^14^. Despite this unknown, BMGs exhibit two detracting features for possible use as a replacement for steel in a flexspline: (1) lower fatigue limits than steel and (2) lower fracture toughness than steel^16^. The hallmark of the SWG is the ability to survive repeated elastic deformation of the steel flexspline while simultaneously transmitting torque without fracturing. The literature on BMGs shows a large variance in the reported values for fatigue and fracture toughness^16^. Early Zr-based BMGs were measured to have a fatigue endurance limit of about 8% of their yield strength (compared to more than 60% in steels like 304 L and 15–5PH) and a fracture toughness less than 20 MPa m^1/2^ (compared to steel with values >100 MPa m^1/2^). Although many new BMGs have been reported with higher toughness and fatigue resistance (up to 55% of their yield strength), steel is normally used as a benchmark against which BMGs are compared^17^.

While this may appear to make BMGs unattractive for SWGs, the fact that the displacement of a flexspline is fixed, not the load, means that the stress experienced in the flexspline is dependent on the Young’s modulus of the material. The lower modulus results in a lower stress in the flexspline, which imparts a higher predicted fatigue life. For example, the strain in a flexspline when expanded with the wave generator is fixed for any specific SWG and can be calculated using an elastic bending argument. The radius of curvature of an ellipse, *R*, is given by


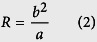


where *b* and *a* are the minor and major axes of the ellipse, respectively. The strain in the flexspline, *ε*_*x*_, can be calculated from


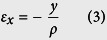


where *y* is the half thickness of the flexspline wall and *ρ* is the distance from the center point of the flexspline to the neutral axis of the flexspline wall. The strain of the flexspline can then be calculated by measuring the change in the curvature before and after the insertion of the wave generator. For a 20 mm diameter flexspline (CSF-8) and a 50 mm diameter flexspline (CSG-20), the strains in the flexspline are calculated to be 0.038% and 0.058%, respectively, using the minimum wall thickness at the root of the teeth. Since the geometries of the flexsplines are fixed, the stress, *σ*_*FS*_, can be calculated for any desired material using the Young’s modulus and Hooke’s Law


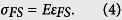


For a stainless steel flexspline (with Young’s modulus of 200 GPa), the elastic stress from inserting the wave generator is estimated to be 8 and 12 MPa, respectively for the small and large flexsplines. For a well-known commercial BMG, LM1b (Zr_44_Ti_11_Ni_10_Bu_10_Be_25_), the calculated stress for the small and large drives is lower at 3.6 and 5.5 MPa, respectively, due to the much lower Young’s modulus of 95 GPa. For a fatigue failure, the stress-amplitude (the ratio of loading stress to yield strength) must exceed the fatigue endurance limit (the maximum stress to survive 10^7^ cycles). Using literature values for both 304 L and 15–5PH stainless steel, it is estimated that the steel flexsplines are being elastically loaded between 1–3% of their fatigue endurance limit for the small flexspline and 1–5% for the large flexspline. In contrast, due to their low modulus and their high strength, the BMG is estimated to be elastically strained to 0.4–2.5% of its fatigue endurance limit for the small flexspline and 0.5–4% for the large flexspline, using the large spread of reported fatigue values from literature. This analysis shows that BMG flexsplines should experience a similar or longer fatigue life than steel versions due to their higher elasticity and yield strength, even though BMGs are inherently more brittle. However, the analysis also shows that if the flexsplines were only loaded elastically by the wave generator, failure via fatigue would never occur (the bearings in the wave generator would always fail first). Since the plot in Fig. 1d shows that fatigue of the flexspline is the primary failure mode at high load torques, the stress on the flexspline from the load transfer in the gear teeth is much higher than simply the elastic expansion from the wave generator.

## Results

To evaluate the potential, we performed a feasibility study to see if BMGs could be successfully manufactured into flexsplines from a variety of different alloys. Two BMG alloys were used for the prototyping, a Zr_35_Ti_30_Cu_8.25_Be_26.75_ (GHDT) known for its high toughness and a new Ti-based BMG, Ti_40_Zr_20_Cu_10_Be_30_, which is a low-density alloy (4.8 g/cm^3^) with a high glass forming ability (~16 mm)^18^. This alloy was based on two previously developed BMG metal matrix composites, DV1 (Ti_48_Zr_20_V_12_Cu_5_Be_19_), and V0 (Ti_53_Zr_27_Cu_5_Be_15_)^19^,^20^. To create the Ti-based BMG used here, the vanadium was removed (which was initially added to composites to provide a beta stabilizing effect) and the titanium was replaced with beryllium (to reduce the volume fraction of the second-phase microstructure and to create a monolithic glass). New alloy variants were created by attempting to decrease the percentage of dense elements, such as zirconium and copper, while maximizing the low-density element beryllium. The process was stopped at the composition Ti_40_Zr_20_Cu_10_Be_30,_ where the density could no longer be lowered without the critical casting thickness of the alloy decreasing below the minimum required to cast the flexspline prototype shown in Fig. 2. The new alloy was characterized using DSC and X-ray diffraction as compared to several other BMGs and the hardness and elastic constants were also measured. The DSC curve demonstrates the Ti-BMG does not have as wide of a supercooled liquid region as GHDT and shows some evidence of crystal phases forming in the prototyped flexspline, but the alloy is still remarkable for its low density and high glass-forming.

**Figure 1:**
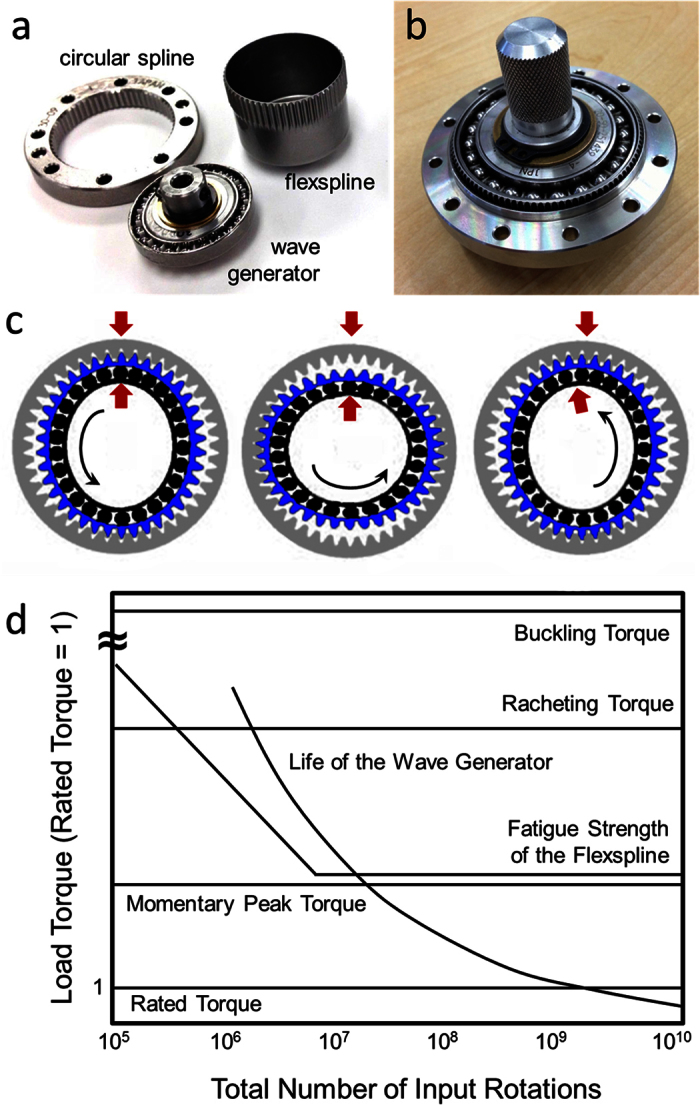
Operation of a strain wave gear (SWG). (**a**) A disassembled SWG showing the three components: an outer spline, a wave generator, and a flexspline. (**b**) An assembled CSF-8 flexspline from Harmonic Drive, LLC. (**c**) A schematic showing the operation of a SWG where each 180° revolution of the wave generator moves the flexspline by one tooth. (**d**) A schematic of a load torque versus number of cycles plot for a SWG showing the various failure mechanisms and how to design for them.

**Figure 2:**
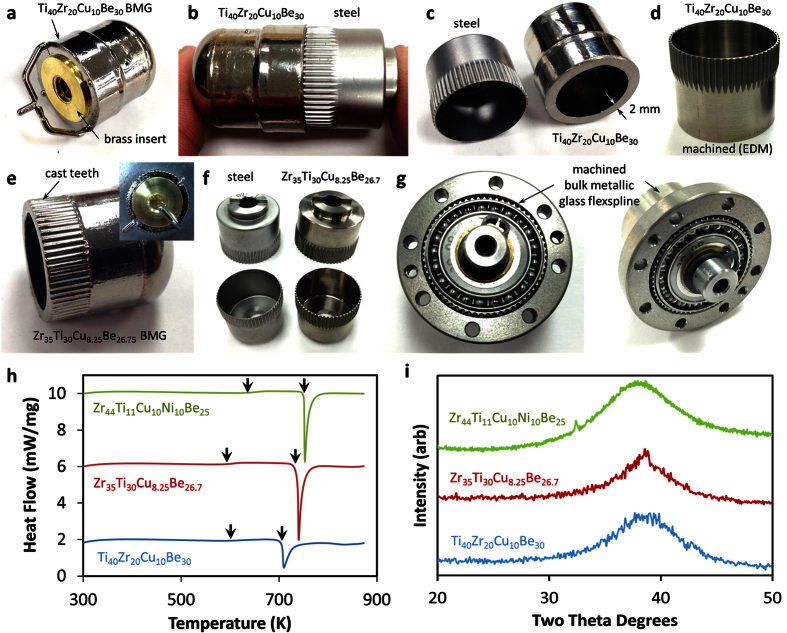
Prototyping bulk metallic glass flexsplines. (**a**) Suction casting over brass inserts was used to create a cup from a Ti-based BMG. (**b**) Comparing the outer shape of the BMG cup to a machined steel flexspline. (**c**) The minimum thickness of the cup using suction pressure was 2 mm. The wall was thinned via conventional machining. (**d**) EDM was used to machine the teeth in the flexspline resulting in the final shape, shown. (**e**) Attempts were made to cast the teeth of the flexspline using and EDMed mold, shown in the inset. (**f**) Comparison of fully prototyped BMG flexspline with a steel version. (**g**) An assembled, functioning SWG utilizing a BMG flexspline from (**f**). (**h**) DSC trace of three BMG alloys cast into flexsplines. The lower two plots were prototyped using lab-grade material while the other plot is from a commercially cast part. (**i**) XRD scans of three BMG alloys cast into flexsplines, showing mostly amorphous microstructure even in fairly large parts.

Table 1 demonstrates that BMGs have typically more than double the strength of crystalline steel and titanium but with much lower Young’s modulus, making them highly elastic. They are also extremely hard, have exceptionally high specific strength for metal alloys and have a low liquidus temperature. These properties demonstrate that BMGs are high-strength, flexible metal alloys that are able to be cast at low temperatures, similar to aluminum. Initially, we attempted to replicate the 20 mm diameter (CSF-8) flexspline using a combination of casting and machining. This was done by fabricating BMG cups with the same outer dimensions as the steel version but with a thicker wall, due to limitations in suction casting pressure. Figure 2a shows the strategy, where the Ti-based BMG was cast over a brass insert using a suction-casting arc melter. A mold was designed with multiple vents, shown in Fig. 1a, which allowed the liquid to completely flow around the insert without flow lines. Once the insert was removed, a BMG cup with a 2 mm wall thickness and the same outer dimensions as the steel flexspline was obtained, see Fig. 2b. Once the preform in Fig. 2c was fabricated from the BMG, the wall was thinned through conventional machining under heavy coolant to the final thickness of ~0.35 mm. A solid model of the steel flexspline was constructed using a parametric model which was compared and adjusted to the actual teeth profile using a microscope so that wire-EDM (electric discharge machining) could be used to replicate the teeth geometry, shown in Fig. 2d.

In addition to wire-EDM for the fabrication of the gear teeth, we also directly cast the gear teeth against a mold that had previously been made using wire-EDM, shown in Fig. 2e for Zr_35_Ti_20_Cu_8.25_Be_26.75_. Using both techniques, we were successful at fabricating BMG flexsplines with very similar dimensions to standard steel flexsplines (the measured dimensions are shown in Table 2). Figure 2f shows the top and bottom of the first BMG flexspline prototype compared directly to the steel version. After manufacturing, the flexsplines were integrated into a CSF-8 SWG using the standard steel outer spline and wave generator. Figure 2g shows the BMG flexspline from Fig. 2f installed in the SWG. A video showing the operation of this flexspline in the SWG is shown in the Supplementary Material. Figure 2(h,i) shows differential scanning calorimetry (DSC) traces and X-ray diffraction (XRD) traces for three BMG flexsplines after manufacturing. The alloy Zr_35_Ti_30_Cu_8.25_Be_26.75_ (GHDT), is known for its high toughness and large thermoplastic forming region (which can be seen in the DSC image as the distance between the arrows indicating the glass transition temperature and the crystallization temperature). The Ti-based BMG (Ti_40_Zr_20_Cu_10_Be_30_) and the Zr-based BMG (Zr_44_Ti_11_Cu_10_Ni_10_Be_25_, LM1b)^21^ have smaller thermoplastic forming regions but are both shown to be mostly amorphous. Figure 2i shows XRD traces from flexsplines made from all three BMGs, showing mostly amorphous microstructures. The LM1b flexsplines are shown later in the text and were commercially cast, showing only small evidence of partial crystallization.

**Figure 3:**
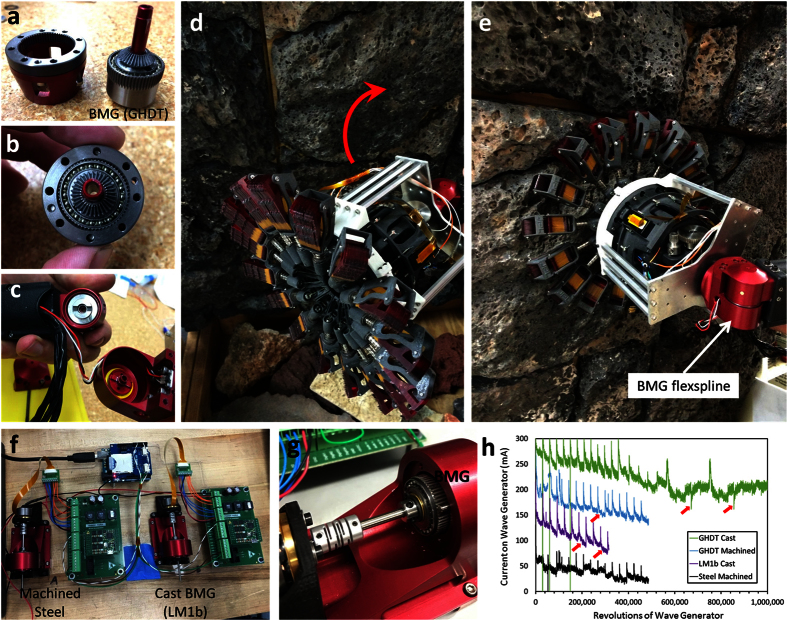
Testing the BMG flexspline in a robotics application. (**a**–**c**) Assembling the BMG flexspline into a SWG used for the joint of the robot. (**d**,**e**) JPL’s wall gripping robot moving to engage a wall using the BMG-containing SWG. (**f**) A side-by-side setup to fatigue flexsplines while measuring the input current. (**g**) Enlargement of the setup showing a BMG flexspline being fatigued. (**h**) A plot of current versus number of rotations of the wave generator for a steel flexspline, a BMG LM1b that was commercially cast, a BMG GHDT that was machined and one that was commercially cast. The red lines indicate locations where cracks were observed.

To demonstrate the load-bearing operation of the BMG flexspline, the part shown in Fig. 2 was installed in NASA JPL’s microspine, wall-gripping robot, shown in Fig. 3^22^. The robot grips by engaging hundreds of sharp metal microspines with imperfections in rough terrain. The 5 kg arm of the robot is positioned by one CSF-8 SWG, shown in Fig. 3. The hybrid steel/BMG SWG was loaded into the robot arm and then used to lift the arm approximately ten times. Figure 3(d,e) shows the BMG SWG rotating the robot arm to engage the rock wall. Video of the SWG operating the robot arm is shown in the Supplementary Material.

Normally, a SWG is characterized by developing stress-life curves on a number of drives until the failure mechanisms are characterized. This would include measuring the ratcheting torque and the efficiency of the drive. Unfortunately, due to the relatively few BMG flexsplines that we were able to prototype, this type of testing has not yet been completed. Instead, a testing rig was built to characterize the fatigue life without any torque being transmitted. Figure 3(f,g) shows the setup where motors drive side-by-side flexsplines at a variable rate while the current is measured. Several BMG flexsplines were run in parallel with steel flexsplines until cracking was observed by a sharp decrease in the current. Figure 3g shows a plot of current versus revolutions of the wave generator for a steel flexspline, a commercially cast BMG LM1b flexspline, a commercially cast BMG GHDT flexspline and a machined BMG GHDT flexspline. The tests were normally run to 0.5 M revolutions, by which point most of the LM1b flexspline will have developed cracks. The flexsplines were fatigued at a high velocity of 3,000 rpm to hasten failure, pausing every ten minutes to allow the motor to cool and to apply a momentary torque to the flexsplines to simulate stopping and starting. The plot shows a noticeable decrease in the current in the LM1b flexspline at approximately 225,000 revolutions. A small crack between the teeth was visible. The test was repeated on a machined GHDT flexspline created from laboratory-grade material and a similar cracking was observed at 300,000 revolutions. Tests on commercially cast GHDT flexsplines showed improved fatigue life, with visible cracks normally appearing beyond 600,000 revolutions. Owing to the lack of outer spline in these tests, the flexsplines did not exhibit the normal compressive forces provided by the outer spline during operation. The cracking of the BMG flexspline was not expected, based on the fatigue analysis provided earlier. One possibility is that a machining phenomenon known as “quilting” introduced pre-cracks in the thin region between the gear teeth, as is shown in the Supplementary Material. Future work will involve testing fully assembled SWGs where no machining has been done on the BMG flexspline after casting. This will require a modified wave generator or modified casting dimensions.

In addition to fatigue testing, hardness and elastic constants were measured for two BMG alloys manufactured in various ways. Table 2 shows properties of Zr_44_Ti_11_Cu_10_Ni_10_Be_25_ (LM1b) and Zr_35_Ti_30_Cu_8.25_Be_26.75_ (GHDT) manufactured into using both laboratory grade material into a plate and using commercial material into a flexspline. Also, in the case of GHDT, a commercially cast flexspline is also compared with a prototyped flexspline using laboratory-grade material. Based on the literature, BMG mechanical properties within the same compositional family can be assessed by measuring the hardness, the Young’s modulus and the shear modulus. The properties of BMGs are highly processing dependent and changes to oxygen content, impurities and cooling rate can profoundly affect them. Typically, the higher the hardness and stiffness of a BMG, the better the wear performance but that comes at a cost in decreasing toughness, which can often be assessed by evaluating the shear modulus, *G*, which represents the barrier to shear flow in BMGs and is directly related to toughness^23^. Table 2 shows that the nominal properties of both BMGs are achieved using laboratory grade material when a 3 mm plate is cast. These parts have a high cooling rate, due to the small casting thickness and have been measured to have less than 50 ppm oxygen. The nominal properties of GHDT and LM1b are relatively similar with the exception of the much higher hardness in LM1b and indeed this alloy has superior wear resistance than GHDT in standard wear tests. When commercial material is used to cast the flexspline, using alloy with 100–150 ppm oxygen, The hardness, Young’s modulus and shear modulus all increase, indicating that the combination of slower cooling rate in the larger part coupled with higher oxygen content has created a modified BMG alloy. In particular, the shear modulus of LM1b and GHDT increased 6% and 8%, respectively, when commercially manufactured. This indicates that these flexsplines will likely be harder and more brittle than laboratory-grade material cast into 3 mm thick plates and indeed, some cracking is observed during testing. GHDT was also prototyped into a flexspline using laboratory-grade material and was conventionally machined using wire EDM. In this sample the shear modulus increased by 5% over the nominal sample and the hardness increased by 2%. This demonstrates that the difference between the properties of laboratory-cast plates and commercially cast flexsplines is a function of both the manufacturing and the oxygen content, which is the expected result.

**Figure 4:**
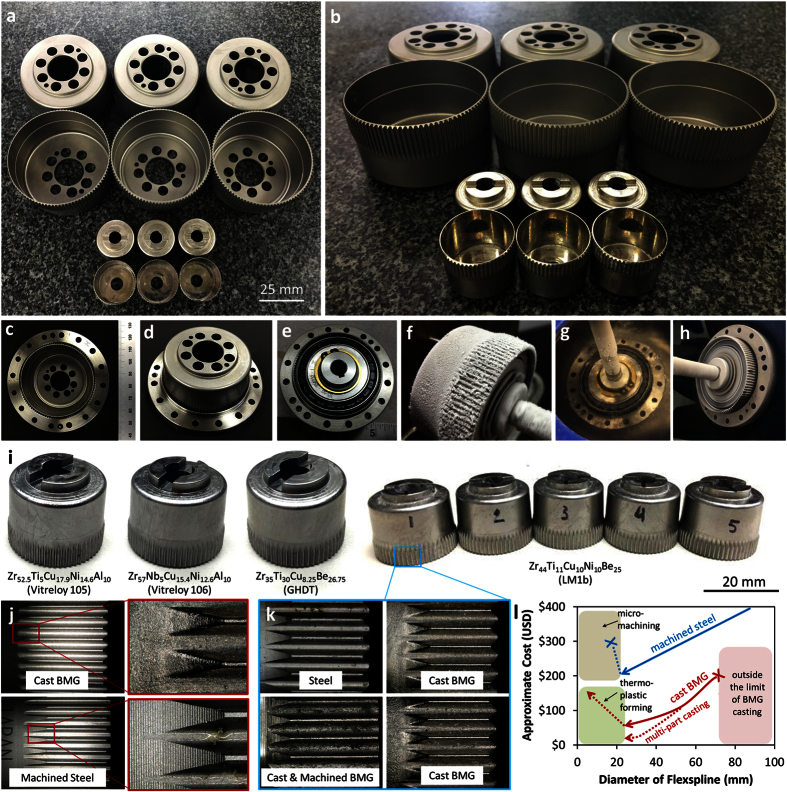
Commercial casting of BMG flexsplines. (**a**,**b**) 50 mm and 20 mm diameter BMG flexsplines cast to near net shape from the alloy LM1b. After casting, the samples have been de-gated and holes machined into the bottom. (**c**-**d**) Inserting the BMG flexspline into a commercial steel outer spline. (**e**) A fully assembled hybrid CSG-20 SWG with a BMG flexspline. (**f**) A still from a video of the BMG flexspline being driven by the wave generator after submersion in liquid nitrogen. (**g**,**h**) Testing a fully assembled SWG with a BMG flexspline at liquid nitrogen temperatures. (**i**) Example of different BMG alloys cast into flexsplines. In total, four alloys were fabricated commercially, as shown. (**j**) Optimal micrographs from the larger 50 mm diameter flexspline comparing the steel part to the cast BMG part. (**k**) Images of 20 mm diameter flexsplines from machined steel, machined BMG and two cast BMG. (**l**) A schematic showing approximate cost associated with machining steel flexsplines and casting >10,000 BMG flexsplines. BMGs can be cast down to ~20 mm in diameter before thermoplastic forming techniques must be used to achieve micro-sized flexsplines. Multi-part casting is possible at small flexspline dimensions.

Next, we attempted to commercially manufacture BMG flexsplines using injection-molding technology available in industry. The CSG-20 and the CSF-8 were the two sizes selected for manufacturing as a way to demonstrate the smallest commercially available cup-type SWG as well as a larger, common size used in robotics. A campaign of casting was performed in collaboration with Visser Precision, Denver CO, to produce near-net shaped flexsplines using Zr_44_Ti_11_Cu_10_Ni_10_Be_25_ (LM1b), a BMG which is the commercial standard for casting. Initial attempts at casting, which are shown in the Supplementary Material, demonstrate that trying to replicate the exact geometry of the machined steel flexspline was not quite possible using existing injection-molding technology. Early attempts to fill the part resulted in turbulent flow, cracking, underfilling and misshapen teeth. It was also very difficult to remove the casting insert. To overcome this problem, minor modifications to the flexspline geometry were implemented, including a slightly thicker wall and a draft angle. This allowed the BMG to fill the mold and the insert to be removed. The thickness of the wall was cast with a thinner step under the teeth to accommodate the wave generator. Figure 4 shows six of the more than sixty successful casts of both the 50 mm and the 20 mm diameter flexsplines. The holes in the base of the flexspline used to transmit torque were machined after casting. The parts shown in Fig. 4(a,b) have only been de-gated, drilled and, in some cases, sand-blasted. After casting, the dimensions of the flexsplines were carefully measured using a variety of techniques and are shown in Table 3. This includes measuring the dimensions of the part, performing optical microscopy to characterize the quality of the casting and profilometry to measure the tooth profile and the surface roughness. Although the molds were created with models developed to exactly to replicate the steel flexsplines, solidification shrinkage and other manufacturing factors resulted in initial parts with small variance in dimensions. After one iteration in the casting, the parts shown in Fig. 4(a,b) were produced. Table 1 shows the properties of the all the BMG alloys that were prototyped into flexsplines as part of the current research. Table 3 shows the variance in the as-cast dimensions of the cast flexsplines and the machined steel versions for several BMG alloys. The demonstrated part-to-part variance in the casting was exceptionally low at 12.7 μm and it is estimated by the commercial vendor that a part tolerance of 6 μm is achievable through casting. It should be noted that the wall thickness of the cast BMG parts is larger than the machined versions due to limitations in the casting.

Figure 4(c,d) shows the assembly of an as-cast LM1b BMG flexspline into a CSG-20 steel outer spline. The as-cast teeth of the flexspline fit well into the outer spline but the inner diameter of the cast part was still slightly oversized for the commercial wave generator. Therefore, the wall was ground down slightly until the wave generator fit, see Fig. 4e. After assembly, the SWG was tested numerous times to transmit torque to the outer spline. A rough test of the function of the hybrid SWG at liquid nitrogen temperatures (76 K) is also shown in Fig. 4. There is a strong desire to develop SWGs that can operate for relatively short lifetimes but at temperatures as low as 70 K without any lubricant as heating liquid lubricants to their operational temperatures may not be possible due to a shortage of available power. It has been shown that BMGs exhibit a linear decrease in toughness as a function of temperature but do not become severely embrittled at temperatures as low as 100 K^24^. To demonstrate basic functionality of the BMG flexspline at these temperatures, Fig. 4(f–h) shows cryogenic operation of the BMG flexspline. Initially, the wave generator was soaked overnight in acetone to remove any lubricant from the ball bearings and then it was inserted into the BMG flexspline. Both were submerged and held in liquid nitrogen and then quickly removed and placed in a jig to lock the flexspline from rotating. The wave generator was rotated with a handheld drill and the flexspline was flexed continuously from 100–300 K as it heated. Figure 4f is a still image from a video shown in the Supplementary Material. Next, the outer spline of the SWG was attached and the entire assembly was tested cryogenically, see Fig. 4(g,h). This demonstrates that, to first order, the BMG flexspline is able to flex and transmit torque at temperatures as low as 76 K with no liquid lubricant. Further testing in an environmental chamber is being planned for future work.

In addition to the CSF-8 BMG flexsplines that were cast, shown in Fig. 4(a,b), several other alloys were also manufactured into these flexsplines to investigate the castability and properties of different BMG alloys. Figure 4i shows the four different BMGs that were commercially cast and their properties appear in Table 1. Two well-known non-Be alloys were cast into the flexspline, Vitreloy 106 (Zr_57_Nb_5_Cu_15.4_Ni_12.6_Al_10_) and Vitreloy 105 (Zr_52.5_Ti_5_Cu_17.9_Ni_14.6_Al_10_). Both alloys formed an amorphous part but their higher melting point and higher viscosity produced a lower quality casting. The BMG GHDT, which was used for the prototypes shown in Figs 2 and 3, was also cast into a flexspline to compare the commercial casting process with the prototype made at JPL using lab-grade material. Lastly, the alloy LM1b was used predominantly for testing and characterization of the casting process. Figure 4(j,k) shows a selection of optical micrographs from both machined steel and cast BMG flexsplines. A Keynance large depth-of-field microscope was used to perform both optical imaging and profilometry on the surface and the gear teeth. Figure 4j shows a comparison between the CSG-50 flexspline cast from BMG and machined from steel. The teeth have virtually the same profile but the surface of the BMG has a random texture compared to the steel, which exhibits horizontal machining lines. The tips of the teeth in the BMG alloy is rounder than the steel, due to the high viscosity of the BMG during casting. Figure 4k shows the smaller CSF-8 flexspline in steel, a BMG prototype that was created using wire EDM and two BMG cast parts. Due to the smaller feature size compared to the larger part, the replication of the gear teeth is not quite as good. The teeth are slightly rounder and the surface is rougher. The BMG sample that was cut using wire EDM shows a very rough surface on the gear teeth as compared to the cast samples, indicating that casting provides a better finish, and likely, improved wear performance. Measurements of cast BMG gears are shown in Table 3.

## Discussion

The collaboration with industry has demonstrated that current supply chains and manufacturing capabilities are suitable for mass production of BMG flexsplines, although some improvements are still required. With conventional SWGs manufactured from steel, the cost of machining each flexspline stays constant with volume. Manufacturing BMG flexsplines using injection-molding technology allows the cost to decrease as volume increases. Table 4 shows a simple cost-benefit analysis provided by industry for mass producing BMG flexsplines and that is shown schematically in Fig. 4l. The cost of each BMG flexspline is based on its mass, so smaller flexsplines are cheaper to manufacture. Moreover, the cost of BMG flexsplines decreases with increasing part numbers due to the re-use of molding and the economy of scale. In mass production (>10,000 parts), the 50 mm BMG flexspline would cost only 11% of the total cost of a commercial SWG ($94 for each BMG flexspline). The plot shows that BMG flexsplines have the potential to be used in dimensions smaller than approximately 75 mm in diameter, where the mass and diameter approaches the commercial limit. Compared to conventional machining, where the cost of manufacturing the flexspline is almost invariant to its size due to the increasingly thin wall, injection-molding is far less expensive as the diameter decreases because multi-part castings become possible (called “trees”). Below a certain size, the wall of the flexspline becomes too thin to cast or machine and other techniques are required. The schematic is based on mass production, which we assume is more than 10,000 parts being made. At this scale, there is a tremendous cost savings by utilizing BMG flexsplines between 20–75 mm in diameter.

The minimum size of a cup-type flexspline is comparable for both machining steel and casting BMGs. The limit of injection-molding BMGs into the thin wall of the cup is approximately 0.5 mm, which is similar to the wall thickness on the commercial CSF-8 flexspline. However, the machining of steel cannot be significantly decreased due to the ultra-thin wall of the cup at smaller dimensions. Micro steel SWGs must use flexspline rings instead of cups. In contrast, BMG flexsplines have no such manufacturing limit. Using techniques like thermoplastic forming or blow molding, BMG flexsplines could be produced at sizes less than 100 μm, similar to what has been done in the literature for BMG microdevices^25^. Micro harmonic drives are already being explored using sun-gear flexsplines and these new geometries may benefit tremendously from the use of BMGs.

This research demonstrates the feasibility of using BMGs as flexsplines in SWGs, which has the potential to be a major disruption in the way that these unique gears are used for both terrestrial and space applications. This work shows that BMGs, while generally brittle, have the strength and elastic strain limit to transmit torque when used as a flexspline. This was done by prototyping a BMG flexspline and implementing it into a NASA JPL robot, where it operated for a short number of cycles without failure. We also successfully fabricated approximately 60 BMG flexsplines commercially from four different alloys. BMG flexsplines were fabricated from Ti-based alloys with 40% lower mass than steel but with higher hardness. Future work will involve improving the casting procedure so that full life testing and efficiency can be measured on the BMG flexsplines and a cost/benefit analyses can be performed. Due to the high precision of the mating between the steel flexspline and the outer spline, the tolerances must be extremely tight for life testing. The BMG parts, while very close, are not exact replicas of machined steel versions, which prevents most testing other than low-cycle bench-testing. In addition to improving the casting tolerances, future work will explore manufacturing the outer spline and the wave generator using BMGs. Cold operation favors using materials with the same coefficient of thermal expansion and the same properties, which motivates making the entire SWG of BMG. The challenge of producing commercially viable strain wave gears using BMGs will most likely result from the material properties of the BMG. We have shown here that the commercial casting does somewhat degrade the properties of the BMG as compared to nominal samples prepared in the laboratory. Creating new flexspline designs specifically to accommodate the properties and casting limitations of BMGs will be an important step towards improving the performance. The largest unknown trade in the use of BMG flexsplines is in the BMG alloy selection. In the current paper, we have looked only at monolithic BMGs however, ultra-tough BMG matrix composites have also previously been developed. Since the performance of a strain wave gear is a competition between abrasive wear and the fatigue of the flexspline, it is still unknown if BMG alloys should be designed for toughness or wear resistance. Further work is required in this area. Overall, however, this work shows that BMGs are a promising material for use in SWGs and may have the potential to drastically reduce their cost, while not sacrificing performance.

## Methods

The BMG prototypes were prepared starting with >99.9% purity starting materials by arc melting in a Ti-gettered argon atmosphere. The resulting ingots were suction-cast into the shape of the flexsplines and then machined to the final shape conventionally. XRD was performed using Cu Kα radiation on a Philips X-pert Pro to confirm the structure. DSC was performed on a Netzsch DSC. Testing apparatuses and methods are described in the text. Commercially cast samples were first prepared in 15 kg batches through vacuum induction melting using >99% starting materials by Materion, Elmore OH. Commercial casting was done using a vacuum linear injection molding machine at Visser Precision, Denver CO.

## Additional Information

**How to cite this article**: Hofmann, D. C. *et al*. Castable Bulk Metallic Glass Strain Wave Gears: Towards Decreasing the Cost of High-Performance Robotics. *Sci. Rep.* **6**, 37773; doi: 10.1038/srep37773 (2016).

**Publisher’s note:** Springer Nature remains neutral with regard to jurisdictional claims in published maps and institutional affiliations.

## Supplementary Material

Supplementary Information

Supplementary Video 1

Supplementary Video 2

Supplementary Video 3

Supplementary Video 4

Supplementary Video 5

Supplementary Video 6

Supplementary Video 7

Supplementary Video 8

Supplementary Video 9

Supplementary Video 10

## Figures and Tables

**Table 1:** Comparison of properties between two common flexspline steel alloys, a common crystalline titanium alloy, a common crystalline aluminum alloy, and the five bulk metallic glass (BMG) alloys used in the current study.

	Alloy	σ_y_ (MPa)	Hv	*E* (GPa)	*ρ* (g/cm^3^)	σ_y_/*ρ* (MPa/(g/cm^3^))	GFA (mm)	Tg (K)	Tx (K)	Tl (K)
Steel	304 L Stainless Steel	210	159	200	8.0	26	na	na	na	1673
Steel	15–5PH Stainless Steel	963	345	200	7.8	123	na	na	na	1677
Titanium	Ti-6Al-4V Grade 5	880	349	114	4.4	200	na	na	na	1877
Aluminum	6061-T6	106	107	69	2.7	39	na	na	na	925
BMG	Zr_44_Ti_11_Cu_10_Ni_10_Be_25_ (LM1b)	2000	530	95	6.1	328	25	631	751	1000
BMG	Zr_35_Ti_30_Cu_8.25_Be_26.75_ (GHDT)	1800	462	90	5.4	333	15	584	738	1044
BMG	Ti_40_Zr_20_Cu_10_Be_30_ (Ti-based)	2000	465	97	4.8	416	16	592	706	1050
BMG	Zr_57_Nb_5_Cu_15.4_Ni_12.6_Al_10_ (Vitreloy 106)	1800	440	83	6.7	269	10	677	750	1093
BMG	Zr_52.5_Ti_5_Cu_17.9_Ni_14.6_Al_10_ (Vitreloy 105)	1800	474	88	6.7	269	10	683	740	1069

Mechanical and physical properties are listed where σ_y_ is the yield strength, Hv is the Vickers hardness, *ρ* is density, σ_y_/*ρ* is the specific strength, GFA is glass forming ability, T_g_ is the glass transition temperature, T_x_ is the crystallization temperature, and T_l_ is the liquidus temperature.

**Table 2:** Comparison in properties between cast BMG flexsplines compared with nominal, laboratory-grade material cast into 3 mm thick plates.

Name	Alloy	Manufacturing Method	Hv	*E* (GPa)	*G* (GPa)
LM1b	Zr_44_Ti_11_Cu_10_Ni_10_Be_25_	Lab grade material cast plate	530	95.2	35.1
LM1b	Zr_44_Ti_11_Cu_10_Ni_10_Be_25_	Commercial material and cast flexspline	536	100.5	37.3
GHDT	Zr_35_Ti_30_Cu_8.25_Be_26.75_	Lab grade material cast plate	462	90.5	33.4
GHDT	Zr_35_Ti_30_Cu_8.25_Be_26.75_	Lab grade material machined flexspline	466	97.4	36.3
GHDT	Zr_35_Ti_30_Cu_8.25_Be_26.75_	Commercial material and cast flexspline	472	95.1	35.2

In the table, Hv is the Vickers hardness, *E*, is the Young’s modulus, and *G*, is the shear modulus. The samples listed as “lab grade plate” were cast into 3 mm thick plates using alloy with less than 50 ppm oxygen. The alloys listed as “commercial flexspline” were manufactured using alloy sourced commercially with between 100–150 ppm oxygen.

**Table 3:** Measurements of steel and BMG flexsplines that have been machined and have been cast.

Material	Flexspline	# flexsplines measured	ID near teeth (mm)	OD on teeth (mm)	Teeth Thickness (mm)	Mass (g)
Steel	CSF-8 machined (Harmonic Drive)	2	19.70 ± 0	20.60 ± 0	0.45 ± 0	3.18 ± 0.02
BMG	BMG (GHDT) Size 8 machined	1	19.70 ± 0	20.64 ± 0	0.47 ± 0	2.36 ± 0
BMG	BMG (LM1b) Size 8 cast	13	19.13 ± 0.04	20.62 ± 0.01	0.75 ± 0.02	4.59 ± 0.06
BMG	BMG (Vitreloy 105) Size 8 cast	3	19.13 ± 0.03	20.65 ± 0.01	0.76 ± 0.01	5.01 ± 0.05
BMG	BMG (Vitreloy 106) Size 8 cast	1	19.10 ± 0	20.64 ± 0	0.77 ± 0	5.06 ± 0
BMG	BMG (GHDT) Size 8 cast	17	19.12 ± 0.01	20.64 ± 0.01	0.76 ± 0.01	4.09 ± 0.04
Steel	CSG-20 machined (Harmonic Drive)	5	49.07 ± 0.05	51.18 ± 0.04	1.06 ± 0.04	28.11 ± 0.21
BMG	BMG (LM1b) Size 20 cast	5	48.75 ± 0.07	51.20 ± 0.03	1.23 ± 0.03	32.39 ± 0.51

The measurements on the cast BMG parts were taken directly after casting but before any additional wall thinning. The outer diameter (OD) was taken on the outside of the cast teeth and the inner diameter (ID) was taken from the same height on the cup but inside the wall. The teeth thickness is the OD minus the ID.

**Table 4:** A cost estimate *per part* showing the advantages of casting BMG flexsplines compared with machining.

Number of flexsplines	BMG Cast (50 mm)	Steel Machined (50 mm)	BMG Cast (20 mm)	Steel Machined (20 mm)
1	$20,000	$300	$10,000	$200
500	$154	$300	$97	$200
5,000	$131	$300	$82	$200
10,000	$94	$300	$59	$200

Assuming the manufacturing costs for the steel flexsplines are fixed for every part, mass production doesn’t reduce the cost of the flexspline. By casting the BMGs, where a single mold can be reused, there is a benefit for mass production. The cost estimate was provided by Visser Precision, Denver, CO.
