# Supplementary material for: Castable Bulk Metallic Glass Strain Wave Gears: Towards Decreasing the Cost of High-Performance Robotics
Source: Sci Rep. 2016 Nov 24;6:37773. doi: 10.1038/srep37773 (PMC5121900; doi:10.1038/srep37773)
Supplement: Supplementary Information [file srep37773-s1.pdf]

Supplementary Material: Castable Bulk Metallic Glass Strain Wave Gears: Towards Decreasing the Cost of High-Performance Robotics

Douglas C. Hofmann[1,2]*, Raul Polit-Casillas[1], Scott N. Roberts[1], John-Paul Borgonia[1], Robert P. Dillon[1], Evan Hilgemann[1], Joanna Kolodziejska[2], Lauren Montemayor[1], Jong-ook Suh[1], Andrew Hoff[2], Kalind Carpenter[1], Aaron Parness[1], William L. Johnson[2], Andrew Kennett[1], Brian Wilcox[1]

[1]Engineering and Science Directorate, Jet Propulsion Laboratory, California Institute of Technology, 4800 Oak Grove Dr. Pasadena CA 91109

[2]Keck Laboratory of Engineering Sciences, California Institute of Technology, 1200 E. California Blvd., Pasadena CA 91125

*Corresponding author, E-mail address: dch@jpl.nasa.gov (D.C. Hofmann).

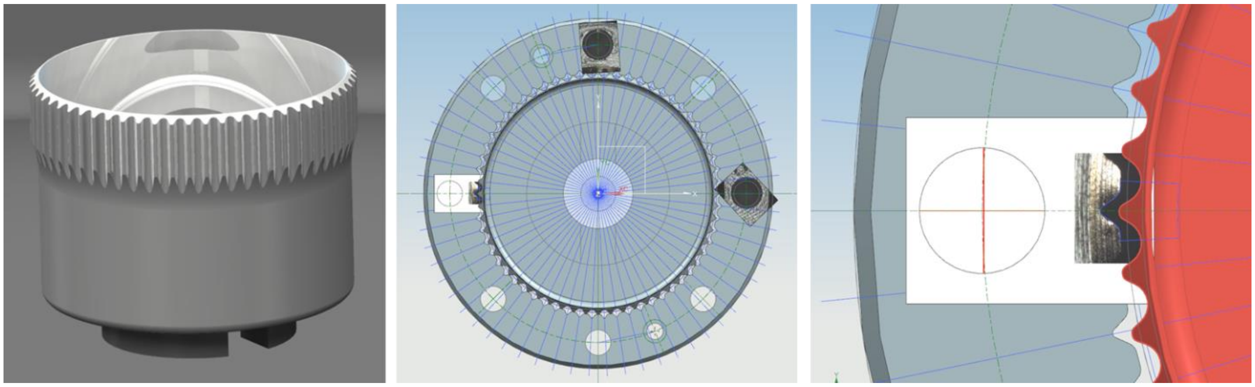

Supplementary Figure 1 – A parametric model was used to create the machining path for the flexspline as well as to create the casting molds which were used for commercial fabrication. In this figure, a rendering of the CSF-8 flexspline is shown along with the model incorporating micrographs taken of a steel flexspline for comparison. The model was fitted to match the steel flexspline, including the shape of the teeth.

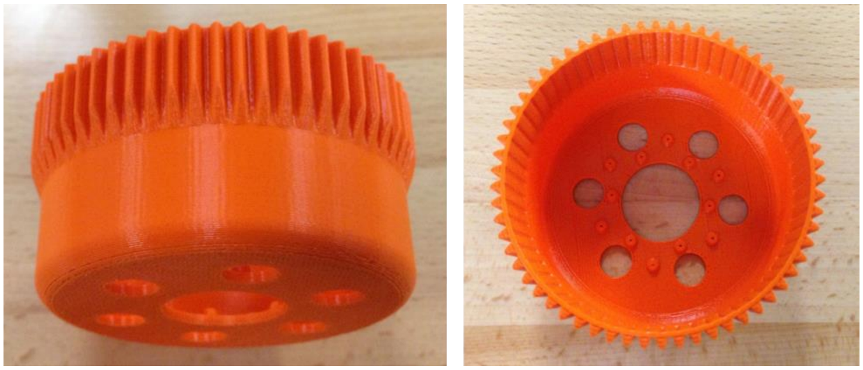

Supplementary Figure 2 – Polymer 3D printing was used to prototype the models created from the flexsplines for fitting purposes. This model shows the CSG-20 flexspline created with ABS plastic.

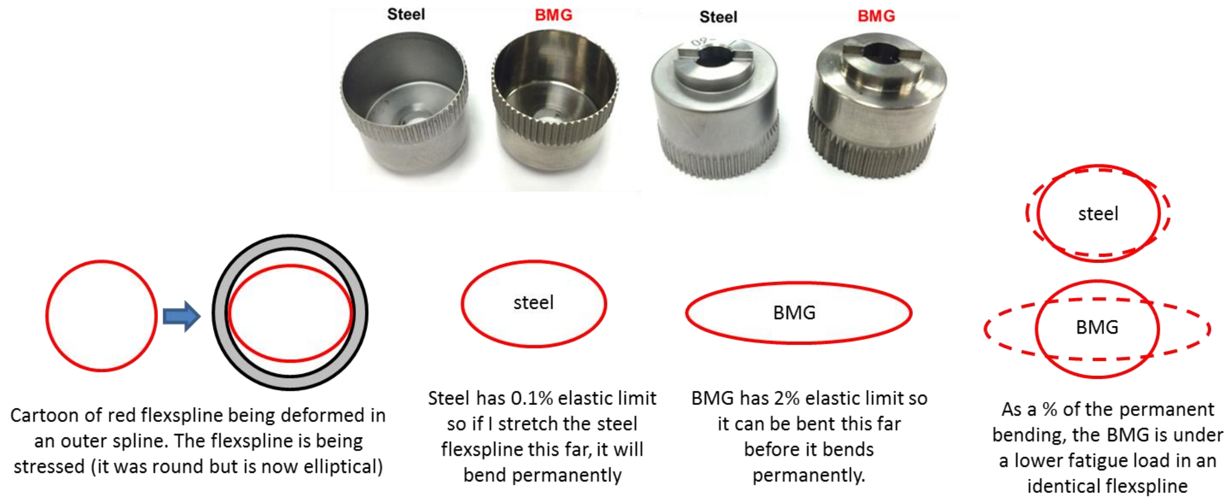

Supplementary Figure 3 – Schematic showing the argument about the loading of the BMG flexspline compared to steel. Since the geometry of the flexspline is fixed, the strain is fixed. This means that materials with higher elasticity are stresses less when integrated into the strain wave gear. This is how a BMG alloy, which is known to be more brittle than steel, can avoid premature fracture due to its lower loading stress.

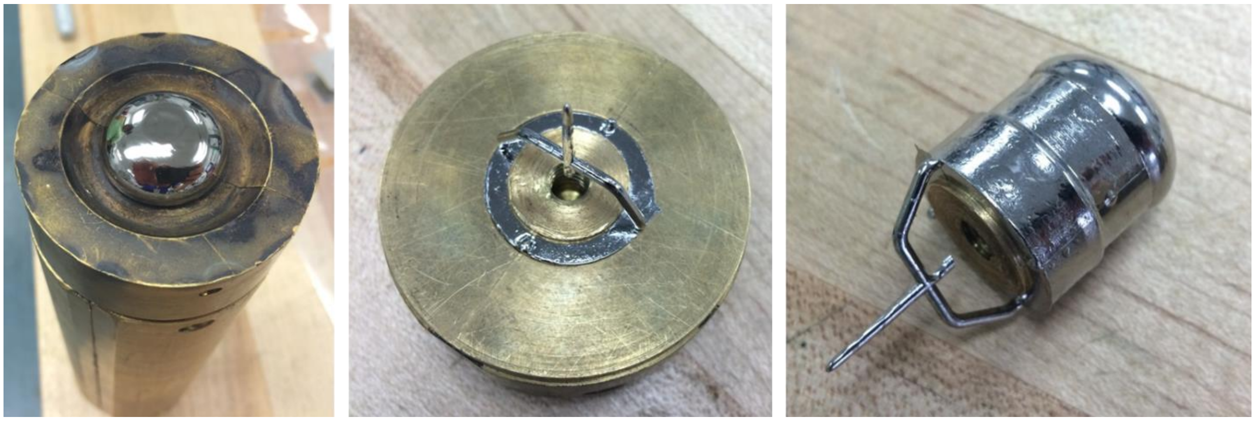

Supplementary Figure 4 – Images of the prototype casting of the BMG flexsplines at NASA JPL. A mold was created with the outer dimensions of the flexspline and the BMG alloys were cast over brass inserts. The final part was then conventionally machined. This technique allowed for the creation of many flexsplines from customized compositions of metallic glass.

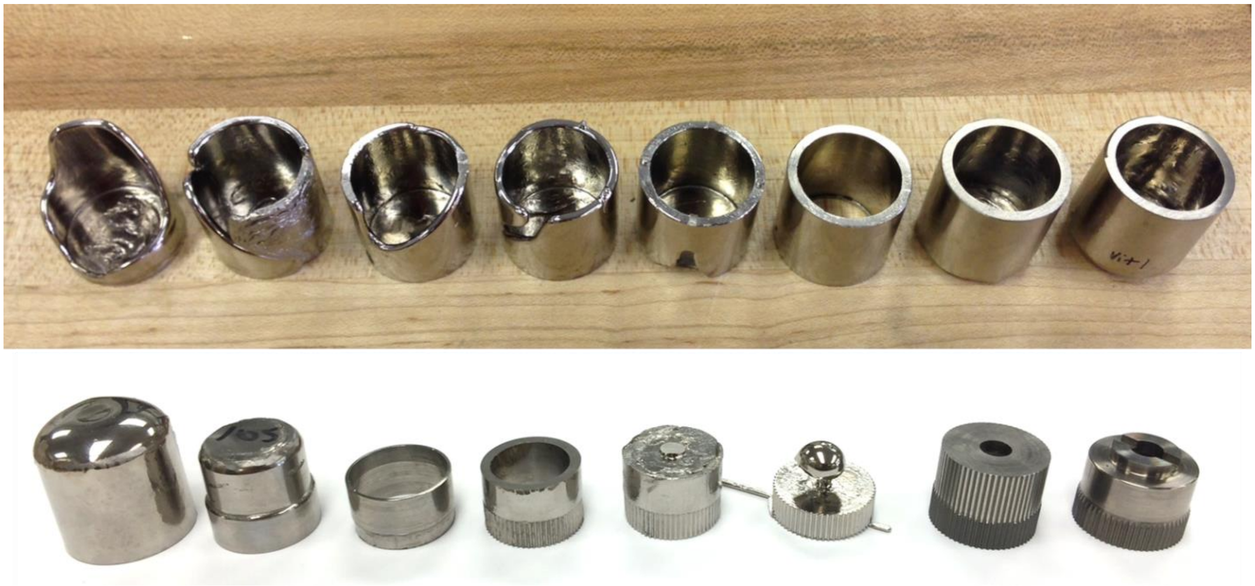

Supplementary Figure 5 – Images showing the prototyping process that was used at NASA JPL to create the blanks needed for the final machining of the flexspline. The upper image shows a progression of learning to cast a cup using BMG. From the left to right, casting procedures were modified to improve flow, which included increasing the cup wall thickness from 1 mm (at the left) to 2 mm (at the right). The lower image shows the progression of casting the cup as well as the teeth of the flexspline from BMG.

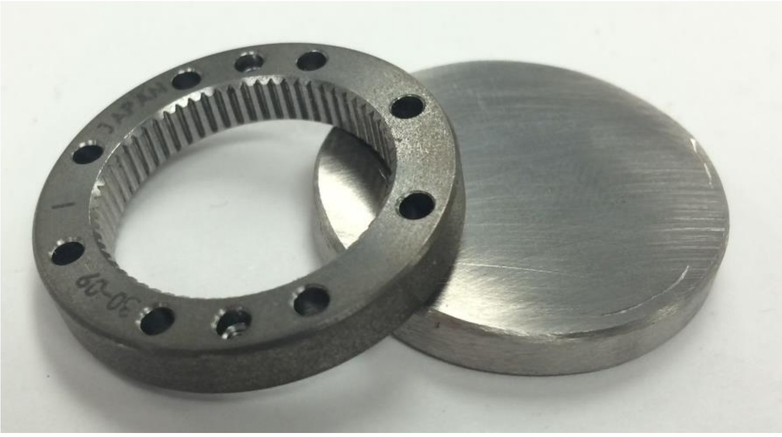

Supplementary Figure 6 – A steel outer spline sitting on a blank of the same outer dimensions cast from a Ti-based metallic glass. Although the primary purpose of the study was on the creation of the flexspline, machining models were also created for the outer spline so that it could be made from metallic glass.

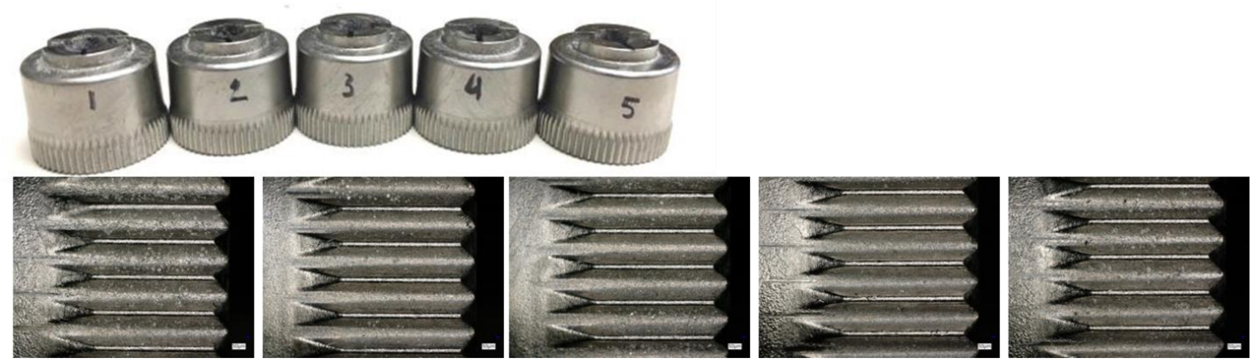

Supplementary Figure 7 – Optical micrographs from the teeth of five cast LM1b metallic glass 20 mm diameter flexsplines. The part-to-part variation was determined to be 12.7 µm.

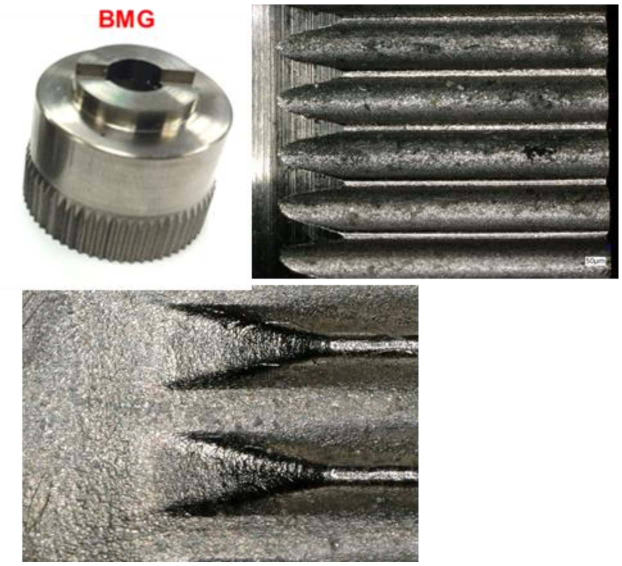

Supplementary Figure 8 – Optical micrographs of a machined BMG flexspline from the alloy GHDT showing a rougher surface compared to the cast components due to the wire EDM.

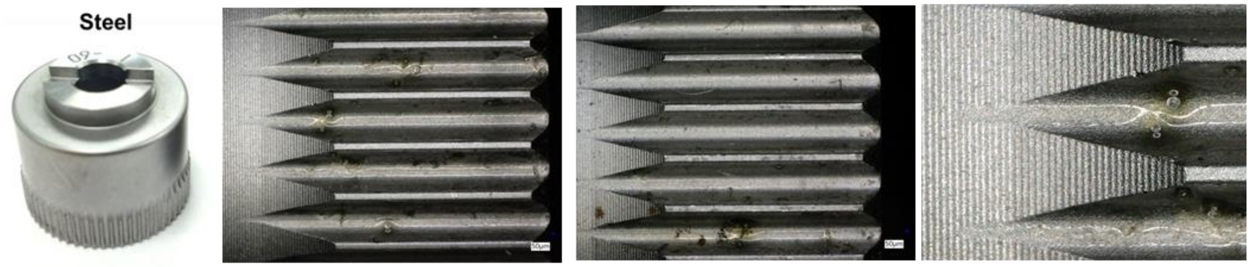

Supplementary Figure 9 – Optical micrographs of a machined steel flexspline showing a comparison between the steel part and the BMG part (shown in the previous figure). The machining marks are visible as well as rust on the flexspline.

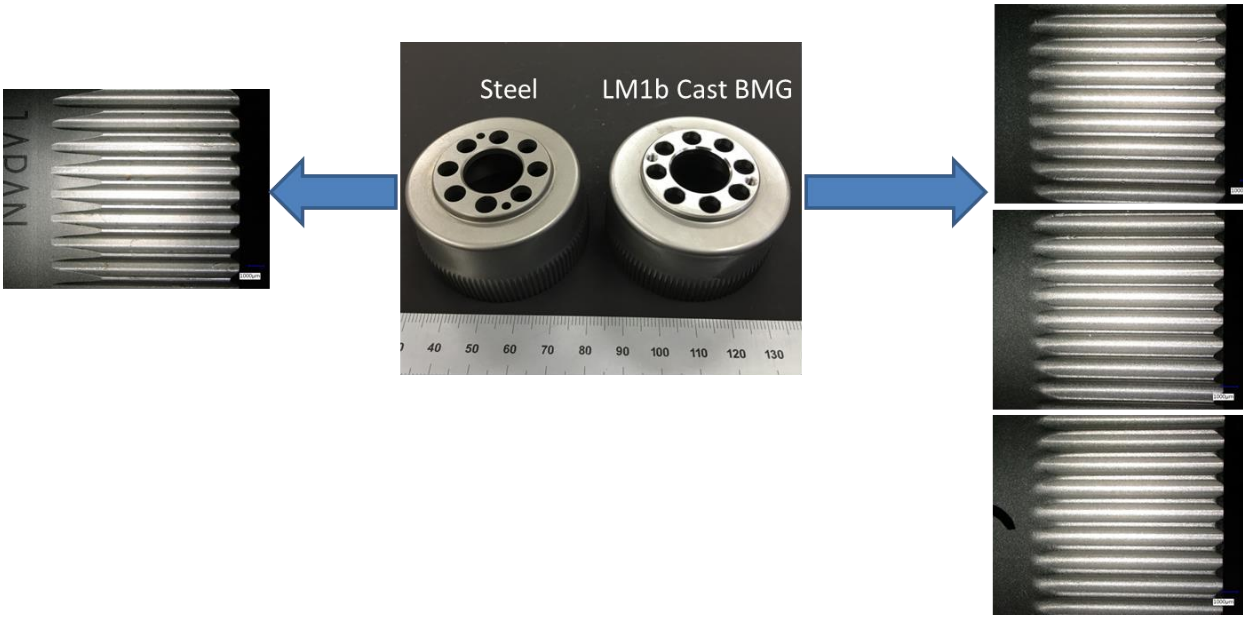

Supplementary Figure 10 – Optical micrographs from the teeth of a 50 mm diameter flexspline showing one steel version (left) and three cast versions from BMG (right). The part-to-part variation in casting is extremely low.

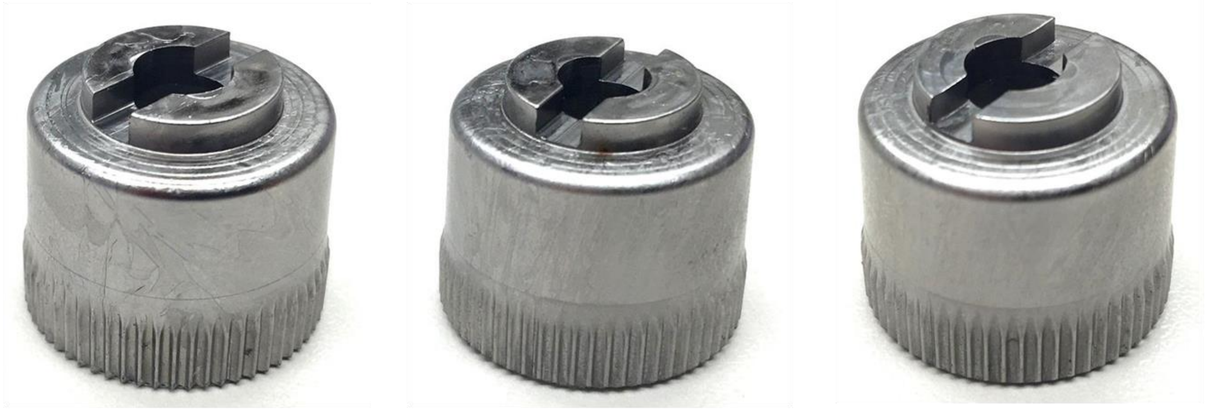

$Zr_{52.5}Ti_5Cu_{17.9}Ni_{14.6}Al_{10}$ (Vitreloy 105)

$Zr_{57}Nb_5Cu_{15.4}Ni_{12.6}Al_{10}$ (Vitreloy 106)

$Zr_{35}Ti_{30}Cu_{8.25}Be_{26.75}$ (GHDT, tough)

Supplementary Figure 11 – Enlarged images of three BMG flexsplines cast with specialty alloys. The two on the left are cast from non-Be Zr-based BMGs, which have a higher melting temperature than Be-bearing alloys. The flexspline on the right is a tough, Zr-Ti-based BMG.

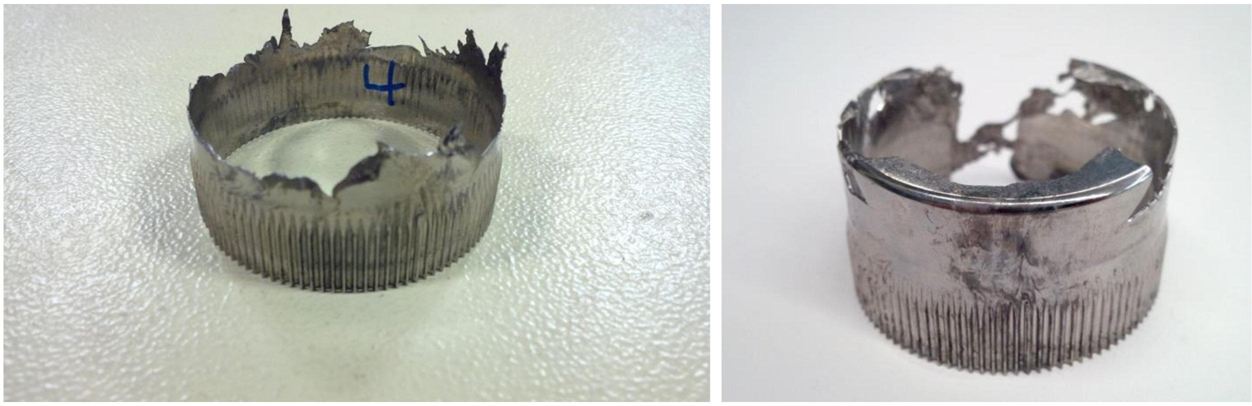

Supplementary Figure 12 – As described in the text, the commercial casting had to go through a few iterations to successfully cast the 50 mm diameter flexspline. These images, provided by Visser Precision, Denver CO, show early attempts to cast the flexspline teeth and wall. In this case, the walls were too thin to allow for full filling of the part.

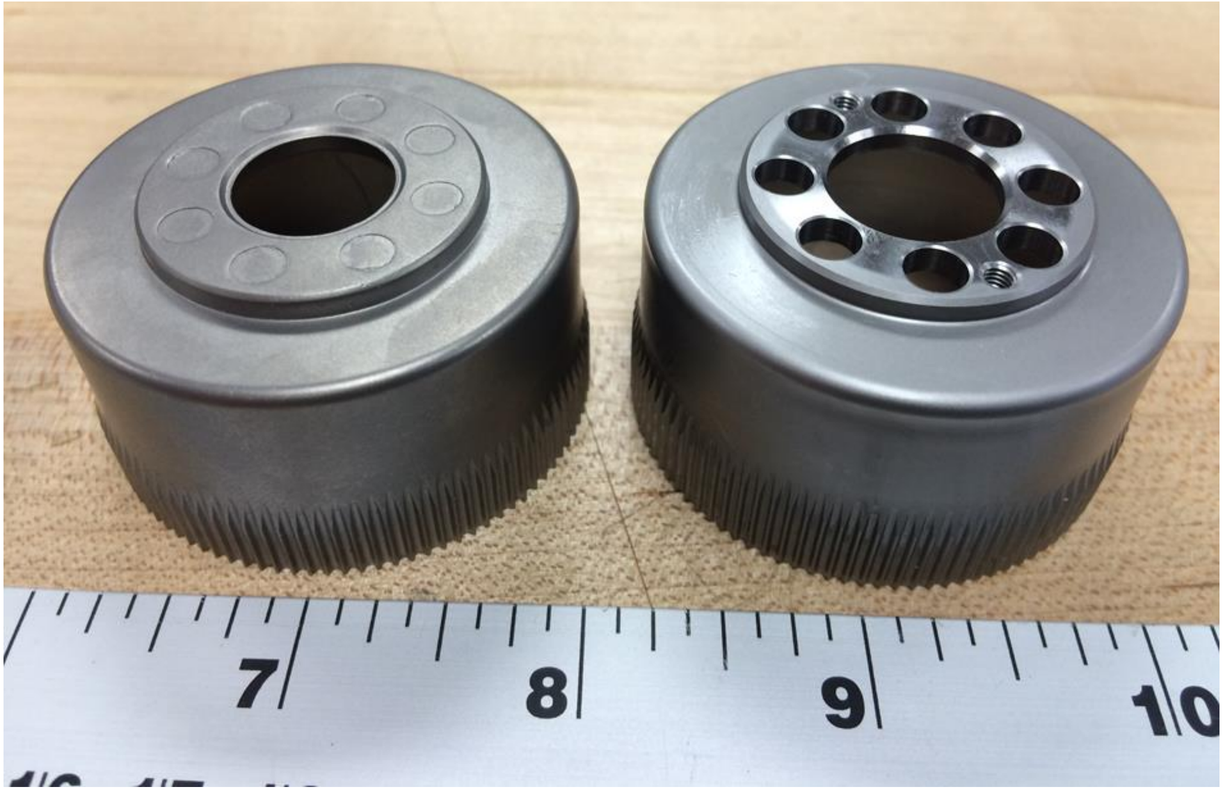

Supplementary Figure 13 – An as cast 50 mm diameter BMG flexspline (left) compared to a machined steel version (right). The location for the drilling of the holes was cast into the part.

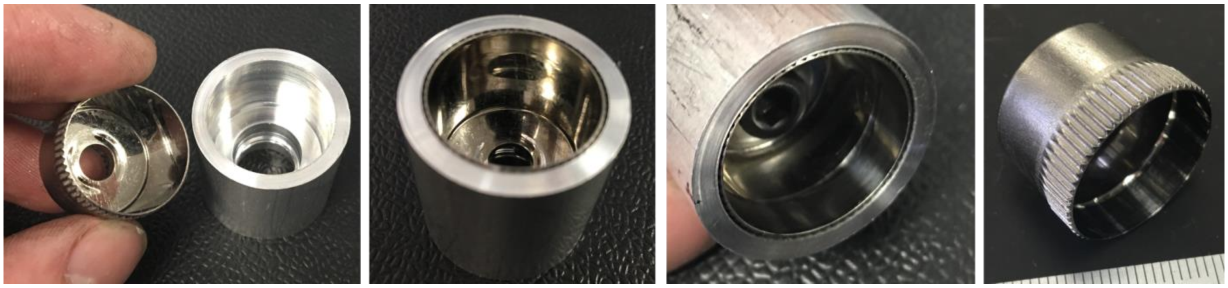

Supplementary Figure 14 – As described in the text, fatigue testing of the flexsplines showed premature cracking, which was most likely caused by machining the cast flexspline walls so they would fit with the commercial wave generators. This figure shows the holder that was used for the machining operation but the "quilting" or "print-through" of the teeth is shown at the image on the right. New strategies are being developed to limit this problem.

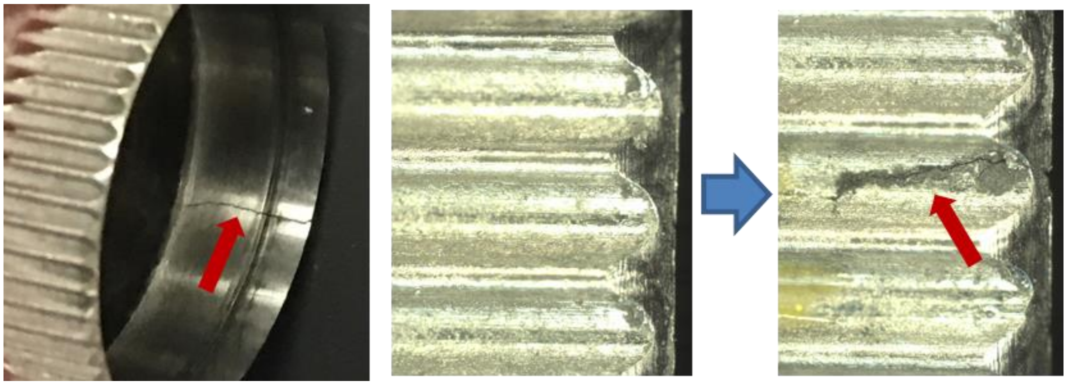

Supplementary Figure 15 – A crack which as developed in a GHDT flexspline after fatiguing it in the gear rig. Optical micrographs show the evolution of the crack from the edge of the cup during the cycling.

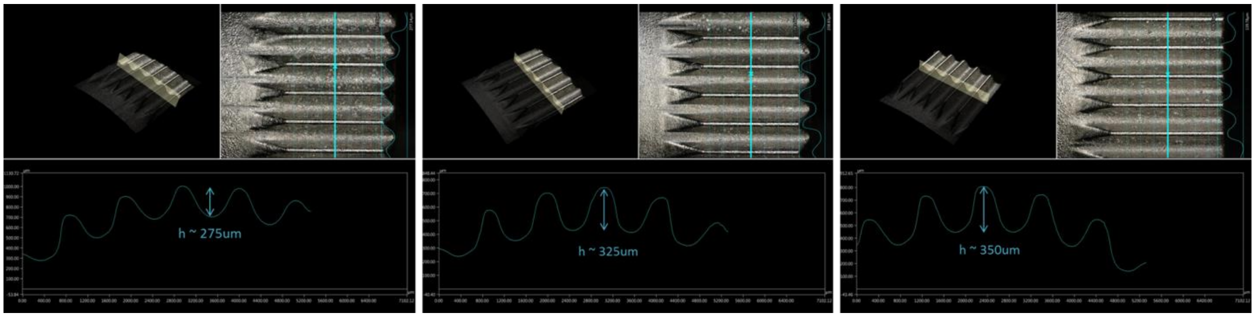

Supplementary Figure 16 – Optical profilometry was used to characterize the casting variance in the teeth of every cast BMG flexspline. For each part, the shape of the teeth was profiled and compared back to the model. The BMG teeth are rounded when compared to machined steel teeth but show low variance between casts.

Supplementary Video Legends

Video 1 - Size 50 BMG Hybrid SWG Bottom View - Operation of a 50 mm diameter strain wave gear with a bulk metallic glass flexspline showing bottom view

Video 2 - Size20 BMG Flexspline Commercially Cast - Flexing of a 20 mm diameter BMG flexspline that has been cast commercially

Video 3 - Size20 BMG Hybrid SWG - Operation of a hybrid strain wave gear with a bulk metallic glass flexspline and conventional steel outer spline and wave generator

Video 4 - Size20 BMG Lifting Robot Arm - JPL wall gripping robot being moved by a bulk metallic glass hybrid strain wave gear

Video 5 - Size50 BMG Flexspline Cold Operation - Operation of a bulk metallic glass-containing strain wave gear after submersion in liquid nitrogen

Video 6 - Size50 BMG Flexspline Liquid Nitrogen Temp - Flexing of the bulk metallic glass flexspline after submersion in liquid nitrogen

Video 7 - Size50 BMG FLexspline Only Side View - Side view of a bulk metallic glass flexspline 50 mm in diameter showing flexing during rotation

Video 8 - Size50 BMG Flexspline Only - Rotation of a 50 mm diameter bulk metallic glass flexspline integrated into a standard steel strain wave gear

Video 9 - Size50 BMG Hybrid SWG Cryogenic Operation - Operation of a 50 mm diameter bulk metallic glass flexspline integrated into a standard strain wave gear after submersion in liquid nitrogen

Video 10 - Size50 BMG Hybrid SWG - Operation of a strain wave gear with a bulk metallic glass flexspline that has been cast commercially
